# Tranexamic Acid in Reducing Gross Hemorrhage and Transfusions of Spine Surgeries (TARGETS): study protocol for a prospective, randomized, double-blind, non-inferiority trial

**DOI:** 10.1186/s13063-019-3231-9

**Published:** 2019-02-12

**Authors:** Shangyi Hui, Liyuan Tao, Feroze Mahmood, Derong Xu, Zhinan Ren, Xin Chen, Lin Sheng, Qianyu Zhuang, Shugang Li, Yuguang Huang

**Affiliations:** 1 Department of Anesthesiology, Peking Union Medical College Hospital, No.1 Shuai Fu Yuan, Wang Fu Jing Street, Beijing, 100730 People’s Republic of China; 2 Research Center of Clinical Epidemiology, Peking University Third Hospital, Beijing, China; 3 Department of Anesthesia and Critical Care, Beth Israel Deaconess Medical Center, Harvard Medical School, Boston, MA 02215 USA; 4 Department of Orthopedics, Peking Union Medical College Hospital, No.1 Shuai Fu Yuan, Wang Fu Jing Street, Beijing, 100730 China

**Keywords:** Topical, Intravenous, Tranexamic acid, Spinal fusion, Blood loss, Non-inferiority trial

## Abstract

**Background:**

Tranexamic acid (TXA) has been routinely delivered in multisegmental spinal decompression and bone graft fusion surgeries with satisfactory outcomes in minimizing gross blood loss and transfusion demands. However, concerns remain that intravenously delivered TXA (ivTXA) may increase risks of postoperative convulsive seizures and systemic thrombogenicity. Topical use of TXA (tTXA), being more targeted to the surgical bleeding site while minimizing patient systemic exposure to ivTXA, has been successfully applied to attenuate blood losses and transfusion requirements in hip and knee arthroplasty. Yet, randomized controlled trials on tTXA efficacy and safety are still lacking in spinal surgeries. With this knowledge gap, we hypothesize that tTXA exhibits non-inferiority to ivTXA in blood conservation and clinical safety in multisegmental spinal decompression and bone graft fusion surgeries.

**Methods:**

A prospective, randomized, double-blind, non-inferiority study design will be adopted. The target sample size is 176. Eligible patients will be randomly allocated to receive either ivTXA or tTXA treatment. The primary end point is the perioperative total blood loss. Secondary end points consist of visible blood losses (intraoperative, postoperative 0–24 h, postoperative 0–48 h, combined perioperative blood loss, total postoperative blood loss), postoperative hidden blood loss, plasma TXA levels, postoperative conventional coagulation monitoring (prothrombin time, activated partial thromboplastin time, fiber Bragg grating, international normalized ratio), postoperative thromboelastography monitoring (reaction time, clot formation time, clot strength, fibrinolysis), postoperative hemoglobin nadir (within postoperative 48 h), perioperative transfusion amounts and rates, and length of hospital stay. Safety end points will be monitored too.

**Discussion:**

This proposed study will contribute to expanding clinical evidences of tTXA for bleeding management in major spinal surgeries. This will be a high-quality prospective randomized trial with sufficient sample size, strict methodology, and few design deficits. It will investigate the potentiality of tTXA as an alternative to ivTXA in improving the current standard of care in multisegmental spinal surgeries, thereby optimizing the enhanced recovery after surgery scheme in spinal surgeries.

**Trial registration:**

ClinicalTrials.gov, NCT03011866. Registered on 5 January 2017.

**Electronic supplementary material:**

The online version of this article (10.1186/s13063-019-3231-9) contains supplementary material, which is available to authorized users.

## Background

Intravenously delivered tranexamic acid (ivTXA) has achieved satisfactory outcomes in minimizing gross blood loss and transfusion demands in complex surgeries [1–3]. However, in recent years, accumulating evidences have questioned the safety of ivTXA, as the treatment has been reported to cause postoperative seizures and systemic thrombogenicity [4, 5]. A 4.1-fold increase of postoperative seizures has been revealed as associated with ivTXA in adult cardiac surgeries [6], with the incidence increasing with larger ivTXA dosages [7, 8]. Additionally, the incidence rate of postoperative thromboembolic events reached 3% after high-dose ivTXA during spinal fusion surgery [9] and 1.7% in cardiac surgeries [4]. Furthermore, ivTXA dose ≥100 mg/kg, age over 75 years, and preoperative renal dysfunction have been identified as risk factors for developing postoperative TXA-related seizures and strokes [10, 11].

As a potential alternative of ivTXA, topical use of tranexamic acid (tTXA) targets concentratively at the surgical bleeding site while minimizing patient systemic exposure to ivTXA. tTXA treatment has been successfully applied to attenuate blood losses and transfusion requirements in hip and knee arthroplasty [12–14]. In spinal surgeries, though, randomized controlled trials (RCTs) on tTXA efficacy and safety are still lacking. With this knowledge gap, we hypothesize that tTXA exhibits non-inferiority to ivTXA in both blood conservation and clinical safety in multisegmental spinal decompression and bone graft fusion surgeries*.* To investigate the research question, a prospective, randomized, double-blind, non-inferiority study design will be adopted.

## Methods/design

### Hypotheses

To investigate the non-inferiority of tTXA to ivTXA in blood conservation and clinical safety, our working hypothesis is that tTXA-treated participants suffer *no more* blood losses or allogeneic transfusions, exhibit *undiminished* postoperative hemoglobin level and coagulation function, and are subject to *no increase* in length of hospital stay (LOH) and adverse event (AE) rate compared with those treated with ivTXA.

### Objectives

With the preceding research hypothesis, the objectives of this study are to compare the perioperative visible/hidden blood losses, transfusion amounts/rates, plasma TXA levels, postoperative hemoglobin level, conventional/thromboelastography (TEG) coagulation monitoring indices, LOH and AE rate between tTXA and ivTXA groups.

### Study setting and investigators

This trial will be conducted at a single tertiary care teaching hospital. The study settings include the wards and the operating rooms of the orthopedic department (200 beds) at this comprehensive hospital (2500 beds). All the laboratory indices in this study will be tested in the key labs of the teaching hospital.

Participants will be recruited through the outpatient clinic of one investigator, and will be consented by the investigators. All the investigators possess working experience in clinical orthopedic research projects. The surgeries will be conducted by a single team of surgeons who have completed clinical fellowship in spinal surgery.

### Design

This is a prospective, randomized, double-blind, head-to-head, non-inferiority trial that conforms to the Consolidated Standards of Reporting Trials (CONSORT) [15].

**Fig. 1:**
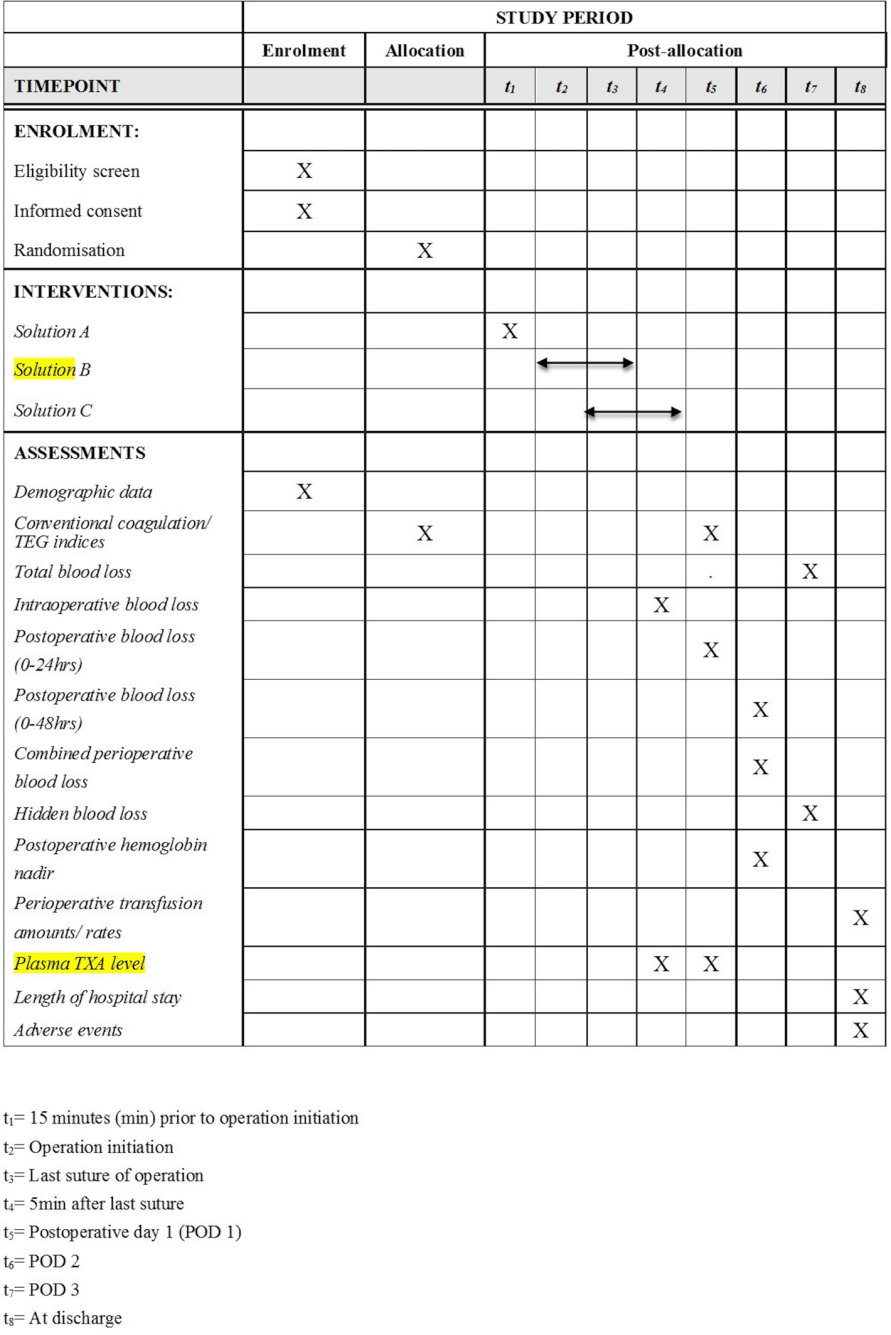
Schedule of enrollment, interventions, and assessments

**Fig. 2:**
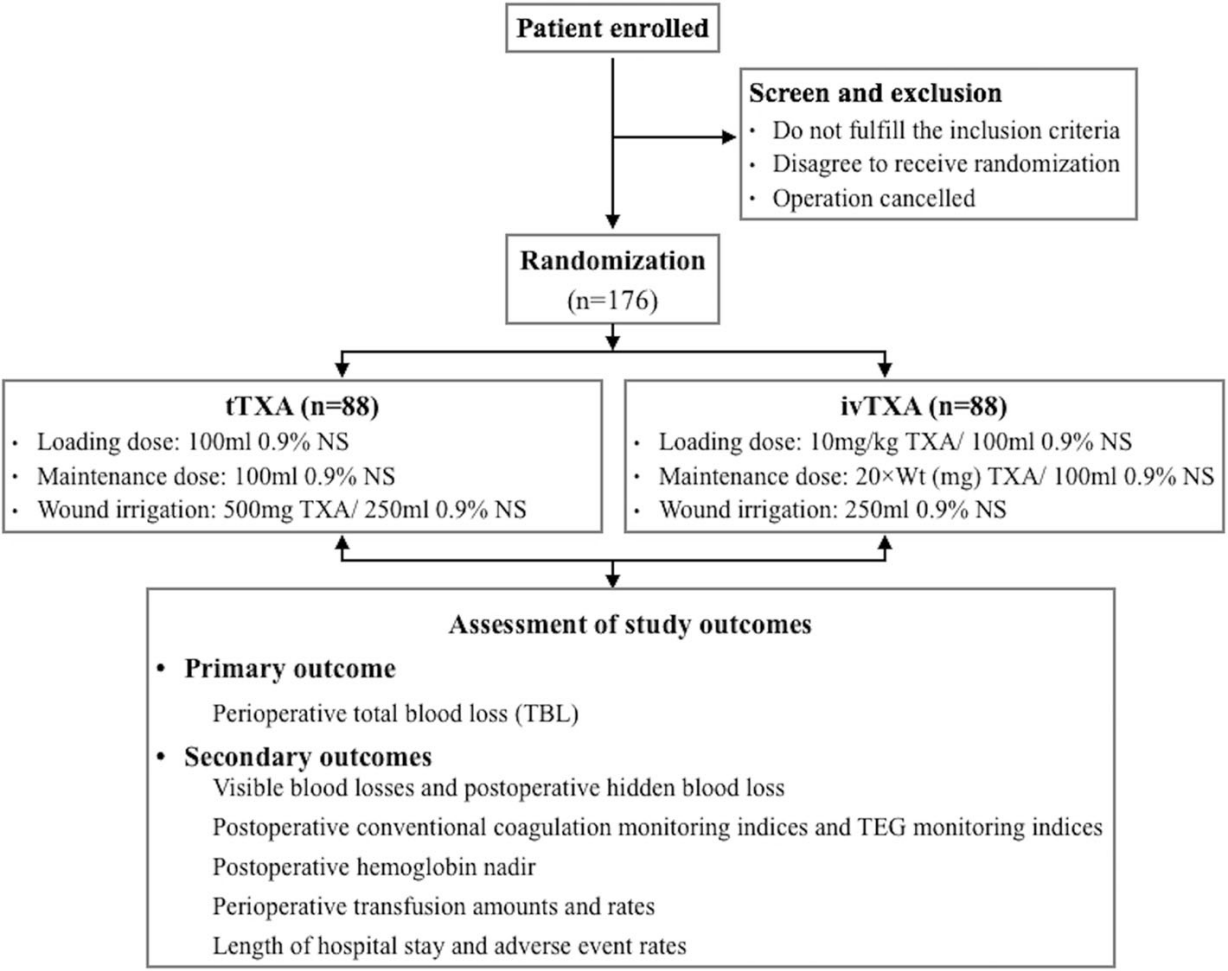
Flowchart of the study process

The timing of interventions and data collection is detailed in Fig. 1. The flowchart of the study process is detailed in Fig. 2. The Standard Protocol Items: Recommendations for Interventional Trials (SPIRIT) checklist is provided as Additional file 1.

### Participants

Patients aged over 18 years who are classified as American Society of Anesthesiologists (ASA) physical status I–II, diagnosed as having lumbar spinal stenosis/intervertebral disc herniation/spondylolisthesis, requiring lumbar spinal decompression and 3–5 levels of bone graft fusion surgeries will be enrolled in the study after signing a written informed consent.

Patients with past medical history of chronic renal dysfunction (preoperative blood creatinine > 120 mmol/L), liver dysfunction (preoperative blood aspartate aminotransferase (AST) or alanine aminotransferase (ALT) > 50 U/L), coronary artery disease with stent placement, abnormal preoperative coagulation profile (preoperative prothrombin time (PT) elongation > 3 s, activated partial thromboplastin time (APTT) elongation >10 s, platelet counts < 100 × 10^9^/L or > 400 × 10^9^/L, or international normalized ratio (INR) > 1.4), or pre-existing anemia (male < 12 g/dL, female < 11 g/dL), and those who have long-term medications of aspirin and/or other anticoagulants, known allergy to TXA, or religious and/or other beliefs limiting blood transfusion are excluded. Participants who undergo dura mater laceration or unexpected massive bleeding or who receive cell saver application during operation will also be excluded.

### Randomization and concealment

Prior to study initiation, an independent researcher who has no contact with any participant will use SAS 9.4 software (SPSS Inc., Chicago, IL, USA) to generate a blocked random number table (block size = 4) to divide eligible participants in a 1:1 ratio into either the tTXA or ivTXA group. Both the participants’ ID numbers and their respective treatment groups (tTXA or ivTXA) will then be sealed in an envelope and made available only to an appointed nurse who is uninvolved in the study conduction.

Participants and researchers will all be blinded to the group allocation. However, at any time point during or after surgeries, any clinician can be “unblinded” to a patient’s treatment group in case of an apparent or suspected medical emergency. A copy of the consent form will be placed inside every chart, and there will also be a note on the front cover of the chart informing that the patient is enrolled in the study, along with contact numbers of the researchers.

### Intervention

On the morning of the surgery, the appointed nurse will prepare Solutions A, B, and C according to the treatment allocation and the patient body weight (Wt, in kilograms) checked with ID number, with 100 ml Solution A as the loading dose, 100 ml Solution B as the maintenance dose, and 250 ml Solution C as the bolus dose. In the tTXA group, Solution A consists of 100 ml 0.9% normal saline (NS), Solution B consists of 100 ml 0.9% NS, and Solution C consists of 500 mg TXA dissolved in 250 ml 0.9% NS. In the ivTXA group, Solution A consists of 10 mg/kg TXA dissolved in 100 ml 0.9% NS, Solution B consists of 20 × Wt (mg) TXA dissolved in 100 ml 0.9% NS, and Solution C consists of 250 ml 0.9% NS. In both groups, Solution A will be infused as a bolus intravenously 15 min prior to operation initiation, Solution B will be infused at 5 ml/h intravenously from operation initiation until the last suture (i.e., 1 mg/kg/h TXA infusion in the ivTXA group), and Solution C will be applied for topical wound irrigation for 5 min with the waste fluid removed by suction before the wound is closed.

### Adverse event management and emergency termination of the study

In this study, AEs include, but are not limited to, deep venous thrombosis, myocardial infarction, pulmonary embolism, cerebrovascular disease, impaired liver function, impaired renal function, incisional hematoma/infection, and convulsive seizures. Patients will be closely monitored during the study period. In case of suspicions for the preceding AEs, the study team will break the blind, and patients will be treated appropriately under the instructions of both spine surgeons and internal medicine physicians. All AEs will be diligently recorded and reported in the final manuscript.

### Outcomes

#### Primary outcome

The primary outcome of this study will be the perioperative total blood loss (TBL), which is the accumulation of the gross blood losses since operation initiation until postoperative day 3 (POD3). TBL is calculated using the formula provided by Gross [16]:

$$ \mathrm{Total}\ \mathrm{blood}\ \mathrm{loss}=\mathrm{PBV}\times \left({\mathrm{Hct}}_{\mathrm{pre}}-{\mathrm{Hct}}_{\mathrm{post}}\right)/{\mathrm{Hct}}_{\mathrm{ave}} $$

where PBV refers to the patient’s blood volume, Hct_pre_ is the initial preoperative hematocrit, Hct_post_ is the hematocrit on POD3, and Hct_ave_ is the average of the Hct_pre_ and the Hct_post_.

PBV is then calculated using the formula proposed by Nadler et al. [17]:

$$ \mathrm{PBV}={\mathrm{k}}_1\times \mathrm{height}{\left(\mathrm{m}\right)}^3+{\mathrm{k}}_2\times \mathrm{weight}\ \left(\mathrm{kg}\right)+{\mathrm{k}}_3 $$

where k_1_ = 0.3669, k_2_ = 0.03219, and k_3_ = 0.6041 for men, and k_1_ = 0.3561, k_2_ = 0.03308, and k_3_ = 0.1833 for women.

#### Secondary outcomes

Secondary outcomes include:

- Visible blood losses (intraoperative, postoperative 0–24 h, postoperative 0–48 h, combined perioperative blood loss) and postoperative hidden blood loss (HBL)
- Postoperative conventional coagulation monitoring indices: PT, APTT, fiber Bragg grating (Fbg), INR, and TEG monitoring indices: reaction time (R time), clot formation time (K time), clot strength (MA), fibrinolysis (LY30)
- Postoperative hemoglobin nadir (within postoperative 48 h)
- Perioperative transfusion amounts and rates
- Plasma TXA levels tested with solid phase microextraction technique (SPME) [18] 5 min after the last suture (t_4_) and on postoperative day 1 (POD1, t_5_).
- LOH

Among the preceding indices, combined visible perioperative blood loss refers to the gross visible blood loss since operation initiation until postoperative 48 h. Two pretrained surgeons will take charge of the visible blood loss assessments. Specifically, for intraoperative visible blood loss, it is calculated by weighing surgical sponges, measuring blood collected by suction canisters, and subtracting all irrigations fluids added to the surgical field. For postoperative visible blood losses, since the drainage fluid in the Hemovac includes not only blood but also tissue fluid exudation [19], the actual postoperative blood loss (PBL) will be calculated using our previously reported formula [20]:

$$ {\mathrm{PBL}}_{24\mathrm{h}\;\left(48\mathrm{h}\right)}=\mathrm{volume}\ {\mathrm{of}\ \mathrm{drainage}}_{24h\;(48h)}\times {\mathrm{Hct}}_{24\mathrm{h}\left(48\mathrm{h}\right)}/{\mathrm{Hct}}_{\mathrm{ave}} $$

where Hct_24h(48h)_ refers to the drainage Hct level tested 24 h or 48 h postoperatively. HBL is also calculated by subtracting the combined visible perioperative blood loss from TBL. All blood losses are determined on the basis of milliliters. If allogeneic transfusion is performed, then TBL is equal to the loss calculated from Hct changes plus the volume transfused.

#### Safety outcomes

The nature, incidence, duration, and severity of AEs, discontinuation due to AEs, AEs occurring during and after intervention discontinuation, body weight, 12-lead electrocardiograms (ECGs), physical examinations, and vital signs will be monitored in this study.

### Data collection and monitoring

Collected data will be entered into electronic case report forms (eCRFs) with double entry and logic validation, and the eCRFs will be uploaded to a central server. To guarantee the quality of trial conduction, a qualified clinical trial expert will be invited to audit the trial implementation process and data entry process every 2 months. The periodic audit is performed to ensure that the protocol and Good Clinical Practices (GCPs) are being followed. The expert may review source documents to confirm that the data recorded on CRFs are accurate. All the study records in the past 2 months will be checked each time to ensure the quality of trial conduct and data consistency between source data and data entered in the database. No interim analysis will be performed in this study.

### Sample size and statistical analysis

Our pilot cohort study indicated expected TBLs of 971 ± 459 ml and 911 ± 460 ml respectively in the tTXA and ivTXA groups in lumbar spinal decompression and 3–5 levels of bone graft fusion surgeries. Using PASS version 11.0, a sample size of 70 in each group is calculated to be sufficient with assumed type I error rate α = 0.05, power of test 1 – ß = 0.90, and 20% of TBL (195 ml) set as the non-inferiority cut off. Taking into account a dropout rate of 20%, a sample size of 88 in each group is eventually determined.

All the randomized participants who signed the Informed Consent Form (ICF) and satisfied all inclusion/exclusion criteria and have completed demographic data will be included in the intention to treat (ITT) analysis set. All the participants in the ITT set who have completed the perioperative TBL will be included in the per protocol (PP) analysis set. All the randomized participants who have received at least one dose will be included in the safety analysis (SS) set. All the analyses on efficacy will be based on the ITT set. Analysis on AEs will be based on the SS set.

For the primary outcome TBL, statistical methods for continuous variable analysis will be used in the non-inferiority test in the ITT analysis set, and a sensitivity analysis will be conducted in the PP set. The missing data will be treated as missing at random, and the multiple imputation procedure will be used for the primary outcome. The difference between the tTXA group and ivTXA group will be reported as mean difference and lower one-sided 95% confidence interval (CI), along with a one-sided *p* < 0.025.

For other outcomes, the Kolmogorov-Smirnov statistic with a Lilliefors significance level will be used for testing normality for continuous variables. Continuous variables with normal distribution will be compared using the Student *t* test between groups. The continuous parameters with a skewed distribution will be tested with the non-parametric Mann-Whitney *U* test between groups. The categorical variables will be presented as frequencies and percentages and compared using the chi-square test or Fisher’s exact test. If the total number of cases is less than 40 or the minimum expected count is less than 5, Fisher’s exact test will be used.

For safety analysis, the AEs and abnormal findings in laboratory tests will be listed with the relationship to the study treatments. Fisher’s exact test will be used to compare the rates of participants who have at least one AE between groups in the SS set.

Statistical analyses will be performed using SPSS version 23.0 software (SPSS Inc., Chicago, IL, USA). A two-sided *p* < 0.05 is considered significant.

### Trial registration, ethical aspects, and informed consent

The study proposal and study materials were approved by the institutional review board (IRB) of Peking Union Medical College Hospital (PUMCH). The study is registered at ClinicalTrials.gov (NCT03011866). When significant modification occurs, we will inform the investigators, all participants, and the trial registry. The investigators will obtain informed consent from each participant before they enter the study. Personal information about the enrolled participants will be safely and confidentially kept, and the anonymized individual patient data will be shared on request.

### Dissemination plan

Results of the trial will be submitted to an international peer-reviewed journal. The results will also be presented at national and international conferences relevant to the subject fields.

## Discussion

It is widely acknowledged that the complexity of multisegmental spinal decompression and bone graft fusion surgery positively correlates with perioperative blood losses. In such surgical arenas, significant blood transfusions are often required, with additional risks of infectious disease transmission, coagulation dysfunctions, and postoperative spinal epidural hematoma formation [21] inevitably introduced. Since ivTXA has been testified as efficacious in reducing blood losses and transfusions in a series of complex surgeries [2, 3, 22], it has also been routinely adopted in spinal surgeries across medical centers.

However, concerns remain over the theoretical thrombogenicity [23] and propensity for convulsive seizures by the use of ivTXA [2, 24]. To settle such concerns, local and more targeted delivery of the drug may provide a promising alternative for blood conservation in spine surgeries while minimizing patients’ systemic exposure to TXA. Currently, the efficacy of tTXA has been well established in major surgeries, especially in hip and knee arthroplasty [12, 14, 25–28]. Yet, clinical research regarding tTXA application in spinal surgeries has been scarce. In a placebo-controlled study, Krohn et al. investigated the impact of tTXA on postoperative blood loss in patients undergoing lumbar instrumented spinal fusion surgery [29]. The wound was irrigated with tTXA for 5 min before removal of excess fluid and wound closure. The postoperative blood loss was reduced by half in patients receiving the treatment, demonstrating the hemostatic efficacy of tTXA. Nevertheless, principal limitations reside in both the quite limited sample size and the placebo-controlled study design; thus, no definite conclusion should be drawn on the comparative hemostatic efficacy of tTXA and ivTXA. Therefore, the present TARGETS study with sufficient sample size will investigate for the first time whether tTXA conveys non-inferior efficacy in managing gross hemorrhage and transfusions compared with ivTXA. With the results of the TARGETS study, we expect to provide high-quality clinical evidence for the role of tTXA in perioperative blood conservation, thereby optimizing the delivery route of TXA in spinal surgeries.

While visible blood losses are well recognized and researched, the invisible HBL is relatively overlooked, largely due to its residual in dead spaces and extravasation into tissues. However, according to previous studies, both ours and others’, quantities of HBL in spine surgeries are substantial. Smorgick et al. reported that HBL accounted for 39–42% of TBL in primary/revision posterior spinal fusion surgeries [30], and a retrospective study conducted at our center reported the percentage as high as 47% [19, 31]. HBL has been reported as significantly associated with increased postoperative complications and LOH [32]; therefore, it constitutes a crucial component of the enhanced recovery after surgery (ERAS) scheme. Since there has been no study investigating the effect of tTXA on HBL, the issue will be investigated for the first time in this TARGETS study, with the hope that the study results will provide solid evidence for constructing the ERAS scheme in the spinal surgery field.

In the present study, thromboelastography (TEG) will be adopted for real-time evaluation of the coagulation system. By utilizing this dynamic functional hemostatic test, alterations in R time, K time, MA, and LY30 during the coagulation and lytic phases can be identified with minimal delay [33]. Combining the point-of-care (POC) TEG results and the systemic TXA levels revealed by the SPME technique [18], the potential rationale of tTXA on perioperative coagulation function will be precisely depicted, thereby attesting to the hemostatic efficacy as well as the safety profile of tTXA.

The strength of this study is that, for the first time, a prospective, randomized, double-blind, head-to-head study design with sufficient sample size will be adopted to investigate the clinical non-inferiority of tTXA to ivTXA in multisegmental spinal decompression and bone graft fusion surgeries. The results of this study will provide high-quality clinical evidence for the hemostatic efficacy and safety of tTXA application, thereby optimizing the TXA delivery route and the ERAS scheme in spinal surgeries.

There are possible limitations of the TARGETS study. Firstly, there may be concerns that wound irrigation with TXA solution might introduce inconsistent drug delivery doses within the surgical site. However, Ker et al. [34] have found that doses of tTXA administered in RCTs were highly heterogeneous (ranging from 0.7 mg to 100 mg/ml of saline solution), while the hemostatic effect was not dose-dependent. Secondly, as per previous studies, both by our team and others [20, 30], Hct level on POD3 is considered to reach a steady state as the patients are assumed hemodynamically stable, and it is thus adopted for calculating TBL. However, both postoperative transfusions and oral intake may vary with each individual patient, which may exert different effects on the Hct levels. To further establish tTXA as a promising blood conservation strategy in spinal surgeries, larger multicenter investigations are still expected.

### Trial status

The study is currently recruiting participants. Important protocol amendments will be communicated to relevant bodies (investigators, IRBs, journals) by Dr. Qianyu Zhuang, trial principal investigator, as soon as changes are made.

## Additional file

Additional file 1: SPIRIT 2013 checklist: recommended items to address in a clinical trial protocol and related documents. (DOC 124 kb)
